# Improved Solid-Contact Nitrate Ion Selective Electrodes Based on Multi-Walled Carbon Nanotubes (MWCNTs) as an Ion-to-Electron Transducer

**DOI:** 10.3390/s19183891

**Published:** 2019-09-09

**Authors:** Saad S. M. Hassan, Ahmed Galal Eldin, Abd El-Galil E. Amr, Mohamed A. Al-Omar, Ayman H. Kamel, Nagy M. Khalifa

**Affiliations:** 1 Chemistry Department, Faculty of Science, Ain Shams University, Abbasia, Cairo 11566, Egypt; ahmeddna2006@yahoo.com; 2 Pharmaceutical Chemistry Department, Drug Exploration & Development Chair (DEDC), College of Pharmacy, King Saud University, Riyadh 11451, Saudi Arabia; malomar1@ksu.edu.sa (M.A.A.-O.); nkhalifa.c@ksu.edu.sa (N.M.K.); 3 Applied Organic Chemistry Department, National Research Centre, Dokki, Giza 12622, Egypt

**Keywords:** nitrate, solid-contact ISEs, SWCNTs, potentiometric sensors, gun powder, wastewater, fertilizer analysis

## Abstract

Possible improvement of the performance characteristics, reliability and selectivity of solid-contact nitrate ion-selective electrodes (ISE) (SC/NO_3_^−^-ISE) is attained by the application of a nitron-nitrate (Nit^+^/NO_3_^−^) ion association complex and inserting multi-walled carbon nanotubes (MWCNTs) as an ion-to-electron transducer between the ion sensing membrane (ISM) and the electronic conductor glassy carbon (GC) substrate. The potentiometric performance of the proposed electrode revealed a Nernstian slope −55.1 ± 2.1 (r² = 0.997) mV/decade in the range from 8.0 × 10^−8^–1 × 10^−2^ M with a detection limit of 2.8 × 10^−8^ (1.7 ng/mL). Selectivity, repeatability and reproducibility of the proposed sensors were considerably improved as compared to the coated disc electrode (GC/NO_3_^−^-ISE) without insertion of a MWCNT layer. Short-term potential stability and capacitance of the proposed sensors were tested using a current-reversal chronopotentiometric technique. The potential drift in presence of a MWCNT layer decreased from 167 μVs^−1^ (i.e., in absence of MWCNTs) to 16.6 μVs^−1^. In addition, the capacitance was enhanced from 5.99 μF (in absence of MWCNTs) to 60.3 μF (in the presence of MWCNTs). The presented electrodes were successfully applied for nitrate determination in real samples with good accuracy.

## 1. Introduction

Nitrates are widely used in different industries such as fertilizer production, drugs, explosives and many other products. These industries significantly contribute to the high concentration of nitrate in sewage [1]. Surface and ground water resources are easily contaminated by nitrate ions, and this causes a serious problem around the world. So, removal of nitrate and detecting its concentration is of great concern. Contamination of water resources with nitrate causes disturbances in the ecological balance which is a hazard to human health. In addition, high nitrate levels cause eutrophication in water bodies, which is manifested through uncontrolled growth of algae [2]. Nitrate can be found naturally in surface water at a level <1 mg/L, but this level is greatly disturbed as a result of the intensive use of fertilizers. The World Health Organization (WHO) has established the maximum allowable nitrate level at 10 mg/L [3,4]. High nitrate concentrations in drinking water is carcinogenic and causes other health problems, such as blue baby syndrome in infants [5]. So, it is of great necessity to present a reliable, selective, sensitive and accurate method for the assessment of trace level concentrations of nitrate.

There are different analytical methodologies for the determination of nitrate that have been reported in the literature such as spectrophotometry [6,7], ion chromatography [8,9], capillary electrophoresis [10], amperometry [11], polarography [12], voltammetry [13], gasometry [14,15], fluorometry [16,17] and atomic absorption spectrometry (AAS) [18,19]. Most of these reported methods are time consuming, need reaction conditions control, complex equipment are required, they suffer from severe interference and are not applicable for samples with a complex matrix [20]. Ion-selective electrodes (ISEs) offer different excellent features, such as miniaturization, ease of measurement and application, rapid analysis with a short response time, simple and cost-effective instrumentation, no sample pre-treatment is required and time-efficiency. In addition, the method is non-destructive and can be applied to turbid and colored samples. Ion-selective electrodes have been widely introduced for the assessment of both inorganic and organic analytes in industrial [21,22], environmental [23,24] and clinical analysis [25,26]. Conventional ISEs that contain an internal solution suffer from difficulties in miniaturization and limited applications. In addition, lower detection limits have been restricted by zero-current trans-membrane ion fluxes [27].

Solid-contact potentiometric ISEs can eliminate the inner filling solution. They are characterized by their convenient storage and maintenance, ease of miniaturization and lower detection limit because of diminished ion fluxes [28,29]. When the metal conductor is directly coated by the sensing membrane (i.e., coated-wire electrodes (CWEs)), the applications of these types of ISEs are very limited because of the lack of long-term potential stability [30]. The addition of an ion-to-electron transducer between the ion-sensing membrane and the electronic conductor increases the potential stability of such sensors. One of these ion-to-electron transducers are carbon nanomaterials. These compounds have attracted a lot of attention due to their unique mechanical, chemical and electrical properties [31], and also have a wide range of applications as an intermediate layer in solid-contact ion-selective electrodes (SC-ISEs); these compounds include graphene, carbon nanotubes, fullrene or carbon black [28,32]. Alhough the usage of nanomaterials improves the analytical parameters of sensors (the potential stability and capacity and the reduction of the resistance of electrodes), they only have a slight effect on the selectivity of the electrodes, which is still comparable to coated-disc electrodes [2].

In this work, we present the analytical parameters of solid-contact nitrate ISEs based on nitron^+^/NO_3_^−^ ion association as a sensory material and multi-walled carbon nanotubes (MWCNTs) as a transducing solid-contact material coated to a glassy carbon (GC) substrate. The introduction of nitron^+^/NO_3_^−^ and MWCNTs significantly improves and enhances the selectivity of the proposed sensors toward NO_3_^−^ ions. The proposed ISE is used for nitrate determination in pharmaceutical formulations, wastewater and fertilizer samples.

## 2. Experimental

### 2.1. Materials

“Nitron (1,4-diphenyl-endoanilino-dihydrotriazole), o-nitrophenyl octyl ether (*o*,NPOE), high molecular weight poly(vinyl chloride) (PVC), tetradodecylammonium tetrakis (4-chlorophenyl) borate (ETH 500) and tetrahydrofuran (THF) were the selectophore reagents obtained from Fluka AG (Buchs, Switzerland). MWCNTs were purchased from XFnano Materials Tech Co., Ltd. (Nanjing, China). All other chemicals used in this work were of analytical-reagent grade. De-ionized bi-distilled water was used to prepare all aqueous solutions. The GC electrode consists of GC rods (2 mm diameter) enveloped in polyetheretherketone (PEEK) polymer bodies and was purchased from Metrohom Instruments (Herisau, Switzerland)”.

### 2.2. Electrode Fabrication

“The GC disc was polished at first with 0.3 µm and 0.05 μm Al_2_O_3_ powder, then washed with water and subsequently cleaned by ultrasonication with water and methanol. The resulting glassy carbon electrode (GCE) was placed into a piece of matched PVC tubing at its distal end. A mixture of 20 mg ETH 500 and 2 mg SWCNTs were spread onto the GC electrode surface and heated by an infrared lamp for 10 s until complete melting of the ETH 500 was achieved. The composite was then left to cool, resulting in a uniform layer attached to the electrode surface. The membrane solution was prepared by dissolving 100 mg of the membrane components in 2 mL THF: nitron^+^/NO_3_^−^ (2 wt %), *o*,NPOE (49 wt %) and PVC (49 wt %). The membrane cocktail (100 μL) was drop-casted onto the transducer layer and left to dry for 2 h. The GC/NO_3_^−^-ISEs without MWCNTs were prepared in the same way. All nitrate electrodes were firstly conditioned in 1 × 10^−3^ M nitrate solution for 1 day and then in 1 × 10^−7^ M NO_3_^−^ for another day”.

### 2.3. Potentiometric and Chronopotentiometric Measurements

“The potentials were measured with the use of an Orion-SA 720 pH meter (Massachusetts, USA). The reference electrode was a double junction, Ag/AgCl filled with 3 M KCl in the inner compartment and 0.1 M LiOAc solution as a bridge electrolyte (type 6.0729.100 Ω, Metrohm, Switzerland). The chronopotentiometry measurements were carried out using a Metrohom potentiostat/galvanostat (Autolab, model 204) purchased from Metrohom Instruments (Herisau, Switzerland) connected to a conventional, three-electrode cell. An Ag/AgCl/3 M KCl/0.1 M LiOAc was the reference electrode, a Pt rod was used as the auxiliary electrode and the SC-ISE was connected as a working electrode. All measurements were performed at 25 ± 1 °C using all solid-state potential ISEs”.

### 2.4. Sandwich Membrane Experiments

“Binding constants of the charged ionophore nitron–nitrate (Nit^+^/NO_3_^−^) ion association complex with nitrate ions were calculated using sandwich membrane experiments [33,34]. In this method, two membranes were prepared individually. One of them had the charged ionophore, PVC and plasticizer, while the other had the same membrane composition but without the charged ionophore. The two membranes were conditioned in 0.01 M NO_3_^−^ solution and each was measured individually in symmetric cell with 0.01 M inner filling and sample solutions. A sandwich membrane was made by pressing the two individual membranes previously prepared together after blotting them individually dry with tissue paper to avoid any aqueous film being present between the two membrane segments. The resulting sandwich membrane was then mounted in the electrode body with the ionophore-containing segment facing the sample solution. The potential difference between the sandwich membrane and the single membrane was used for the stability constant calculation”.

### 2.5. Nitrate Assessment in Wastewater, Fertilizers and Gun Powders

“For nitrate determination in industrial wastewater, 1 L of the sample solution was collected, mixed well and filtered off. A 1 mL portion of the filtrate was mixed with a 9 mL aliquot of 30 mM phosphate buffered solution and the mixture was shaken well”.

“For fertilizer samples, 0.1 g of the nitrate fertilizer was placed in a 100 mL conical flask and mixed with 20 mL 0.1 M K_2_S_2_O_8_ and 5.0 mL 2 M NaOH. The mixture was autoclaved at 120 °C for 30 min as previously described [35]. After sample digestion, the solution was transferred to a 50 mL measuring flask and filled to the top with distilled water. The filtrate was adjusted to pH 5 with a few drops of concentrated H_2_SO_4_. In a 25 mL beaker, a 1 mL aliquot of the sample solution was mixed with 30 mM PBS”.

“Different gun powder samples were accurately weighted, dissolved in 30 mM PBS and filled to the top in a 100 mL volumetric flask. A 1 mL portion of the clear supernatant was diluted to 50 mL with PBS. The proposed electrode was inserted in conjunction with the reference electrode in the above final working solutions and the measured potential was then recorded and compared with the calibration graph”.

## 3. Results and Discussion

### 3.1. Potentiometric Characteristics

**Figure 1:**
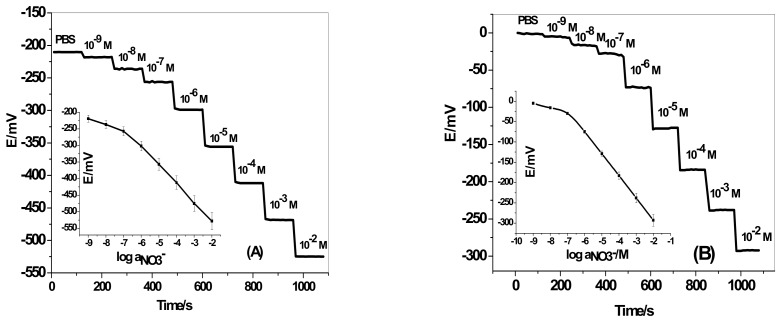
Potentiometric response curves of: (**a**) glassy carbon (GC)/multi-walled carbon nanotubes (MWCNTs)/NO_3_^−^ ion-selective electrode (ISE); and (**b**) GC/NO_3_^−^- ISE in 50 mM phosphate buffer solution, pH 3.5.

**Figure 2:**
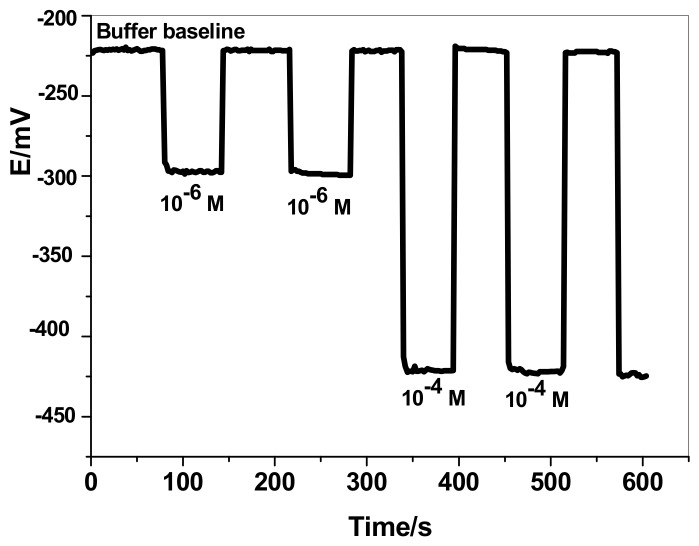
The reversibility test of GC/MWCNTs/NO_3_^−^-ISE.

Solid-contact nitrate sensors based on nitron^+^/NO_3_^−^ ion-association that were plasticized in *o*-nitrophenyloctyl ether (NPOE) were prepared and characterized. The composition of the sensor membrane was: plasticizer = 49 wt %, nitron^+^/NO_3_^−^ = 2.0 wt %, and PVC = 49.0 wt %. The potential of the developed ISEs was recorded over the concentration range 1 × 10^−8^–1 × 10^−2^ M KNO_3_. The potential readings versus log *a_NO3−_* are presented in Figure 1. The slopes calculated from the linear range of the calibration plots of the nitrate-selective electrodes with/without MWCNTs were −55.1 ± 2.1 (r^2^ = 0.9973) and −53.1 ± 1.4 (r² = 0.9995) mV/decade over the linear range of 8.0 × 10^−8^–1 × 10^−2^ and 1.0 × 10^−7^–1 × 10^−2^ M, respectively. The detection limits of these sensors were 2.8 × 10^−8^ (1.7 ng/mL) and 4.7 × 10^−8^ (2.9 ng/mL), respectively. All potentiometric characteristics for all investigated electrodes are presented in Table 1. A stable response was obtained over the pH range 3.5–10. So, phosphate buffer solution (30 mM, pH 5) was chosen for all nitrate measurements. From the presented results, it can be demonstrated that the insertion of a MWCNT layer as an ion-to-electron transducer has no effect on the potential performance characteristics of the solid-contact ISE. As shown in Figure 2, the reversibility test of the studied ISEs is presented. GC/MWCNTs/NO_3_^−^-ISE showed enhanced potential reversibility towards NO_3_^−^ ions. The response time of the GC/MWCNTs/NO_3_^−^-ISE was very short (<5 s). It can be deduced that the removal of the inner filling solution favors the response time of the solid-contact electrode.

### 3.2. Selectivity

The selectivity coefficients of the proposed SC/ISEs were calculated using the modified separate solution method (MSSM) [36]. All anions used in this study were in their sodium or potassium form and the selectivity coefficient values are listed in Table 2. Measurements were carried out in the concentration range of 1×10^−5^ M to 1×10^−2^ M solutions of interfering anions. The plots and Nernstian slopes for the measured ions are shown in Figure S1. It is clear that the nitrate-selective membrane using nitron^+^/NO_3_^−^ as an ion-carrier shows excellent selectivity towards NO_3_^−^ over other anions such as Cl^−^, SO_4_^2−^, S^2−^, F^−^, CH_3_COO^−^, PO_4_^3−^and NO_2_^−^. The proposed ISE revealed high discrimination against these tested anions and allows it to be used to measure nitrate in the presence of a high interference background.

### 3.3. Chronopotentiometry

**Figure 3:**
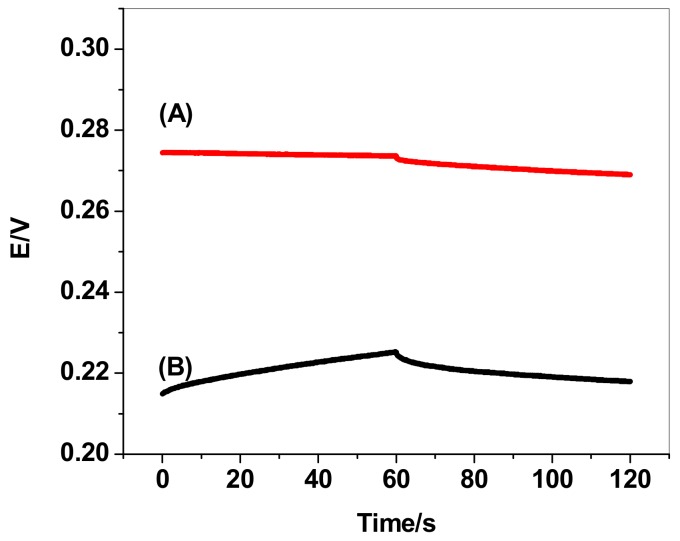
Chronopotentiometry for nitrate-ISEs (**a**) with and (**b**) without MWCNTs as a solid-contact material.

Reversed-current chronopotentiometry was utilized to study and evaluate the short-term potential stability of the GC/MWCNTs/NO_3_^−^-ISE [37]. Typical chronopotentiograms for both GC/MWCNTs/NO_3_^−^-ISE and GC/NO_3_^−^-ISE are shown in Figure 3. From the slope (ΔE/Δt), the potential drift for GC/MWCNTs/NO_3_^−^-ISE was calculated to be 20.33 µV/s, which is much lower than that of the GC/NO_3_^−^-ISE (143.22 µV/s). From these results, it was indicated that the potential stability of the nitrate sensor is enhanced and improved after using MWCNTs as a solid-contact transducer. The low-frequency capacitance for GC/MWCNTs/NO_3_^−^-ISE and GC/NO_3_^−^-ISE is calculated from the equation *Δ*E/*Δ*t = I/C [37] and found to be 49.2 ± 1.3 µF and 6.9 ± 0.8 µF, respectively. From the mentioned results, it was noticed that the nitrate-ISE modified with MWNTs showed significantly lower potential drift and higher capacity in comparison to the coated disc electrode developed under similar conditions. These results confirm the results reported for the successful use of MWCNTS as a transducer material and in anion sensor design [38,39,40,41].

### 3.4. Binding Constants of the Charged Ionophore

The complex stability constant of the nitrate charged-ionophore was measured by the sandwich membrane method. In this method, the membrane potential *E*_M_ is determined by subtracting the cell potential for a membrane without the charged ionophore from that of the sandwich membrane. The stability constant (log *β*) is calculated from the following equation by assuming only one NO_3_^−^ ion binds to one ionophore molecule (*n* = 1):

*E_M_* = *RT/Z_i_F ln* [*β_ILn_*(*L_T_*-*nR_T_*/*Z_i_*)*^n^*]

where *L*_T_ is the total concentration of the charged ionophore in the membrane segment, *R*_T_ is the concentration of lipophilic ionic site additives, *n* is the ion–ionophore complex stoichiometry and *R*, *T*, and *F* are the gas constant, the absolute temperature and the Faraday constant, respectively. The ion *I* carries a charge of *Z*_I_. The potential of the sandwich membrane was found to be −451 ± 6 mV, which gives a stability constant (log *β*) of 8.21 ± 0.06.

### 3.5. Analytical Applications

For the demonstration of the feasibility of the proposed GC/MWCNTs/NO_3_^−^-ISE for environmental analysis, the nitrate content in real wastewater samples containing nitrate ions, commercial ammonium nitrate fertilizers, and gun powders were analyzed by direct potentiometry. A comparison was also made using the standard ion chromatography method [42]. The results obtained by the two methods were in a good agreement within less than ±1.5% as shown in Table 3 and Table 4. An *F*-test showed no significant difference at the 95% confidence level between means and variances of the potentiometric and chromatographic sets of results. The calculated *F* values (*n* = 5) were found to be in the range of 1.24–5.25 compared with the tabulated value (6.39) at the 95% confidence limit.

## 4. Conclusions

A new all-solid-state nitrate-ISE has been developed based on MWCNTs as an ion-to-electron transducer. MWCNTs were revealed to have a large double layer capacitance, fast charge transfer, and high hydrophobicity. The proposed ISE revealed a stable potential response in the linear range of 8.0 × 10^−8^–1.0 × 10^−2^ M with a slope of −55.1 ± 2.1 mV/decade and a detection limit of 2.8 × 10^−8^ M. In addition, the sensor exhibited considerable potential stability and no water film is formed between the sensing membrane and the GC substrate. The sensor is successfully applied for the determination of nitrate in real environmental samples.

## Figures and Tables

**Table 1:** Potentiometric response characteristics of the proposed nitrate ISEs in 30 mM phosphate buffer solution, pH 5.

Parameter	GC/MWCNTs/NO_3_^−^-ISE	GC/NO_3_^−^-ISE
Slope, (mV/decade) *	−55.1 ± 2.1	−53.1 ± 1.4
Correlation coefficient, (r^2^)	0.9973	0.9995
Linear range, (M)	8.0 × 10^−8^–1.0 × 10^−2^	1.0 × 10^−7^–1.0 × 10^−2^
Detection limit, (M)	2.8 × 10^−8^	4.7 × 10^−8^
Working range, pH	3.5–10	3.5–10
Response time, (s)	<10	<10
Life span, (weeks)	8	8
Standard deviation, (mV)	1.3	1.7
Accuracy, (%)	99.3	98.7
Precision, CV_w_, (%)	1.1	0.9

* Average of five measurements.

**Table 2:** Potentiometric selectivity coefficients (*K^Pot^_NO3,B_*) of the proposed SC/ISEs.

Interferent, *B*	log *K^Pot^_NO3,B_*	
	GC/MWCNTs/NO_3_^−^-ISE	GC /NO_3_^−^-ISE
Cl^−^	−5.10 ± 0.4	−5.08 ± 0.2
SO_4_^2−^	−6.50 ± 0.7	−6.60 ± 0.3
S^2−^	−4.70 ± 0.3	−4.64 ± 0.4
F^−^	−6.80 ± 0.9	−6.92 ± 0.1
CH_3_COO^−^	−3.30 ± 0.3	−3.22 ± 0.6
PO_4_^3−^	−7.10 ± 0.2	−7.01 ± 0.2
NO_2_^−^	−4.10 ± 0.3	−4.07 ± 0.6

**Table 3:** Potentiometric determination of NO_3_^−^ in some wastewater samples.

Sample Source	Nitrate-N * (mg/L)		Difference
	Proposed Potentiometric Method	Ion Chromatography Method [42]	
Nitrate fertilizer factory, outfall (I)	38.6 ± 0.3	39.9 ± 0.8	1.3
Nitrate fertilizer factory, outfall (II)	40.3 ± 0.4	42.6 ± 0.3	2.3
Raw sewage plant inflow	110.3 ± 0.9	115.4 ± 0.7	5.1
Aerated lagoon effluent	23.2 ± 0.9	25.6 ± 0.3	2.4

* Average of six measurements.

**Table 4:** Potentiometric determination of nitrate in some fertilizers and gun powders.

Sample Source	Labeled (NO_3_-N)	Nitrate-N *, Recovery%		Difference
		Proposed Potentiometric Method	Ion Chromatography Method [42]	
Ammonium nitrate fertilizer (Alex Fert. Co.)	16.5–17.5% (*w*/*w*)	98.3 ± 0.2	97.5 ± 0.6	0.8
Ammonium nitrate fertilizer (El Naser Fert. Co.)	16.5–17.5% (*w*/*w*)	98.5 ± 0.6	97.8 ± 0.3	0.7
Gun powder 1	-	99.7 ± 0.3	99.5 ± 0.4	0.2
Gun powder 2	-	99.2 ± 0.5	99.6 ± 0.6	0.4

* Average of six measurements.

## Supplementary Materials

The following are available online at https://www.mdpi.com/1424-8220/19/18/3891/s1, Figure S1: The plots and Nernstian slopes for the measured ions using (A) GC/MWCNTs/NO_3_^—^ISE; and (B) GC/NO_3_^—^ISE.
