# Supplementary material for: Improved Solid-Contact Nitrate Ion Selective Electrodes Based on Multi-Walled Carbon Nanotubes (MWCNTs) as an Ion-to-Electron Transducer
Source: Sensors (Basel). 2019 Sep 9;19(18):3891. doi: 10.3390/s19183891 (PMC6766930; doi:10.3390/s19183891)

Figure S1. The plots and Nernstian slopes for the measured ions using (A) GC/MWCNTs/NO_3^-ISE; and (B) GC/NO_3^-ISE.

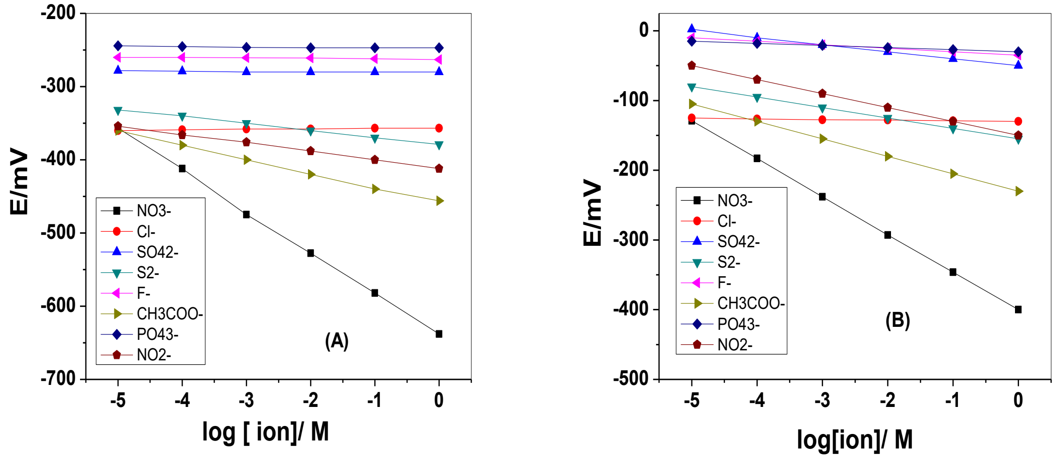

Supplement: Supplementary file 1 [file sensors-19-03891-s001.pdf]
